# Shift work relationships with same- and subsequent-day empty calorie food and beverage consumption

**DOI:** 10.5271/sjweh.3903

**Published:** 2020-10-30

**Authors:** Ting-Ti Lin, Chang Park, Mary C Kapella, Pamela Martyn-Nemeth, Lisa Tussing-Humphreys, Kathleen M Rospenda, Shannon N Zenk

**Affiliations:** 1 School of Nursing, National Defense Medical Center, Taipei City, Taiwan; 2 Department of Health System Science, College of Nursing, University of Illinois at Chicago, Chicago IL, USA; 3 Department of Biobehavioral Health Science, College of Nursing, University of Illinois at Chicago, Chicago IL, USA; 4 Division of Academic Internal Medicine, College of Medicine, University of Illinois at Chicago, Chicago IL, USA; 5 University of Illinois Cancer Center, University of Illinois at Chicago, Chicago IL, USA; 6 Department of Psychiatry, University of Illinois at Chicago, Chicago IL, USA

**Keywords:** circadian rhythm, eating behavior, food consumption, ecological momentary assessment, shift worker

## Abstract

**Objectives:**

Shift work may contribute to unhealthy eating behaviors. However, the evidence is built mainly on comparisons of eating behaviors between shift and non-shift workers. Growing research has suggested daily experiences and exposures may contribute to daily fluctuations in people’s food consumption. The purpose of this study was to examine within-person associations between shift work and same- and subsequent-day empty calorie food/beverage consumption.

**Methods:**

This was a 14-day intensive longitudinal study using ecological momentary assessment. A convenience sample of 80 hospital registered nurses working a rotating shift in Taiwan completed a 21-item food checklist assessing their empty food/beverage consumption (ie, fast/fried food, sweet and salty snacks, sugar-sweetened beverages) four times at random daily. Daily shift work (ie, day, evening, or night shift) was derived from the registry-based work schedule. Three-level mixed-effects regression models were employed for hypothesis testing.

**Results:**

A total of 77 participants with 2444 momentary assessments were included in the final analysis. The results suggested that participants on night compared to day shifts had higher likelihoods of fast/fried food intake [adjusted odds ratio (OR_adj_) 1.7, 95% CI 1.2–2.6] and sugar-sweetened beverage consumption (OR_adj_ 1.5, 95% CI 1.0–2.1). However, there were no significant associations between shift work and subsequent-day empty calorie food/beverage consumption.

**Conclusions:**

Night shift work is associated with same-day increased empty calorie food/beverage consumption among workers. Strategies that help to prevent unhealthy eating behaviors on night shifts may help to reduce rotating shift workers’ empty calorie food/beverage consumption and ultimately improve their health.

To provide around-the-clock services, many industries (eg, healthcare) utilize shift work to extend their operational hours “24/7” (1). In general, shift work refers to work that requires workers to be on duty between 18:00–06:00 hours (2). Globally, shift workers account for approximately 20–25% of the workforce (3, 4). Moreover, in the US, two thirds of shift workers engage in rotating shift work (4), defined as periodically working different shifts (2).

Previous studies have found shift work, particularly rotating shift work, to be associated with increased risk of chronic diseases (eg, type 2 diabetes) (5, 6). Exposure to artificial light at night and changed sleep–wake cycle due to shift work may disrupt workers’ circadian rhythm and adversely affect shift workers’ health. Disrupted circadian rhythm has been linked to desynchronized appetite hormones (eg, decreased leptin) (7), which may increase appetite and food consumption (8). In addition, it has been documented that shift work may contribute to reduced insulin sensitivity (9) and decreased glucose tolerance (10), and in turn increased risk of type 2 diabetes (11, 12).

Empty calorie food/beverage consumption (ie, foods and beverages high in solid fats or added sugars and low in nutrients) (13) has also been linked to an increased likelihood of the aforementioned chronic diseases (14, 15). Shift work may play a role in between-person differences in empty calorie food/beverage consumption (16, 17). Relative to working on regular day shifts, research has found working on non-day shifts (ie, evening or night shifts) was related to higher intake of sweetened foods (17, 18) and sweetened beverages (18, 19). Thus, empty calorie food/beverage consumption, may be one of the pathways by which evening and night shift work increases chronic disease risk (20). Furthermore, research suggested that there may be a joint association of rotating shift work and unhealthy lifestyle (eg, low dietary quality) with increased type 2 diabetes risk (21).

Recent studies have shown that empty calorie food/beverage consumption can vary day-to-day or moment-by-moment based on experiences or environmental context (eg, stress, shift work), suggesting that empty calorie food/beverage consumption may vary not only between but also within persons (22, 23). Additionally, people’s experiences and exposures on a given day may influence their behaviors on the subsequent day (23, 24). Cain et al 2015) observed that participants consumed more high-fat foods on the morning after being exposed to a simulated night shift (24). This indicates that a person’s empty calorie food/beverage consumption may differ depending on their shift work that day or the previous. Yet, no study has examined how changes in work schedule timing relate to within-person daily fluctuations in empty calorie food/beverage consumption.

Building on prior research, the purpose of this study was to examine contributions of shift work to within-person variations in daily empty calorie food/beverage consumption using an intensive longitudinal study design (25) and ecological momentary assessment (EMA). Two hypotheses on within-person associations were tested: (i) on days when working evening or night shifts, empty calorie food/beverage consumption will be higher compared to days when working day shifts, and (ii) on days when working evening or night shifts, empty calorie food/beverage consumption will be higher on the subsequent day compared to days when working day shifts.

## Methods

### Sample

Our target population was rotating shift (ie, a monthly work schedule including at least two shifts timings such as day and night shifts) workers. A convenience sample of registered nurses working in the Taiwan accredited hospitals (26) was recruited using electronic flyers shared on social media platforms (eg, Facebook). Inclusion criteria were as follows: (i) Taiwanese registered nurse working full-time (ie, 40 hours per week), (ii) 20–65 years old, (iii) working on rotating shifts in the hospital for ≥6 months and in the next 30 days, and (iv) no intention to leave the nursing profession in the next month. Exclusion criteria were: (i) having no smartphone or having a smartphone but no access to internet services, (ii) unwilling or unable to provide registry-based work schedule, (iii) working in an administrative position such as Dean or Head Nurse, and (iv) pregnant.

**Figure 1:**
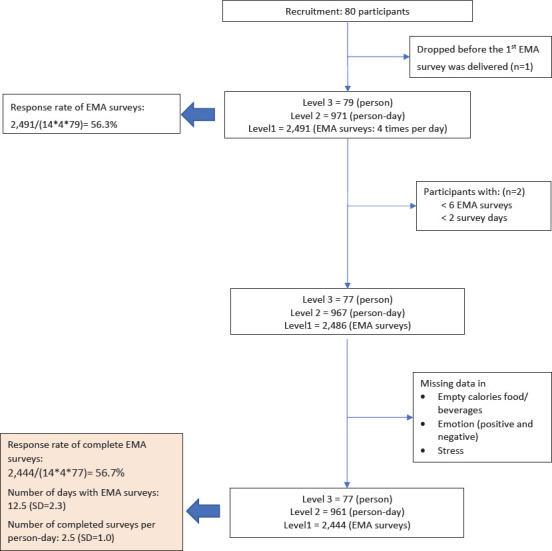
Process of data collection and exclusion.

We recruited 80 registered nurses working in hospitals in Taiwan between October 2018 and January 2019; 1 dropped out of the study before the first EMA survey was sent. Our goal was to preserve as much of the sample as possible, but we recognized some people really did not participate. Thus, we excluded those whose participation was effectively zero. Our main interest was within-person associations between shift work and empty calorie food/beverage consumption. Therefore, we excluded participants with an EMA response rate <10% (ie, <6 completed EMA surveys) or <10% of possible valid EMA survey days (ie, a valid day was considered those in which ≥2 daily EMA surveys were completed) (N=2). As a result, the analytic sample comprised 77 participants. Only EMA surveys with complete data were included in the analysis. The detailed information related to data collection and exclusion is displayed in figure 1.

### Study design and procedure

This was an intensive 14-day longitudinal study (25) using EMA (ie, a research approach that assesses participants’ behaviors or experiences in the natural environment in real-time) (27) with three phases: baseline visit, consecutive 14-day EMA data collection period, and post visit (approximately two weeks after the baseline survey). During the baseline survey visit, we (i) obtained written informed consent; (ii) administered a questionnaire, (iii) provided instructions for EMA surveys, including how and when to answer an online survey in the REDCap system on the smartphones and what to do when there was downtime in this system; and (iv) collected the published work schedule during the past 30 days and the prospective work schedule for the next 30 days.

During the 14-day EMA data collection period, empty calorie food/beverage intake, work schedule, and potential confounders were collected using the EMA surveys. The EMA sampling method can be roughly divided into two categories: event-based sampling (ie, collecting data when an event occurs) and time-based sampling (ie, collecting data over time) (27). In this study, the EMA sampling method was time-based. Participants were signaled to complete the EMA survey using a provided survey link by either text message or e-mail four times per day at random during the following time blocks: 03:00–09:00, 09:00–15:00, 15:00–21:00, and 21:00–03:00 hours. Based on a participant’s anticipated shift schedule and wake–sleep pattern, these four time blocks were adjusted as required. Most survey signals were sent via text message, with a text reminder every 15 minutes if there was no response from the participant. The survey was available for an hour after the initial text message was sent. If the participant did not complete the survey within an hour, the survey was closed and recorded as missing. Due to an unforeseen technical problem in which the messaging system had regular maintenance downtime each day (ie, 13:00–16:00 hours local time), an email survey signal was sent during that period using the Qualtrics system.

During the post visit, we administered a survey related to changes in shift schedules over the prior two weeks and provided compensation for participation of up to approximately US$30 in cash. The Institutional Review Boards (IRB) at the University of Illinois at Chicago (No. 2018-0950) and National Taiwan University Hospital (No. 201712216RIND) approved this study.

## Measures

### Shift work

Shift work was a day-level measure. It was assessed based on the time when a participant started their work shift (ie, day, evening, night) (28), documented on the published work schedule. We operationally defined night shifts as shifts with working hours covering 24:00–08:00 hours.

### Empty calorie food/beverage consumption

Consumption of empty calorie foods/beverages was a moment-level variable. It was assessed four times per day with a 21-item checklist based on the following question: “*Since the last signal, have you consumed or used any of the following items? (Please check all that apply).”* The 21-item food checklist was created based on the top sources of empty calorie foods/beverages reported in the 2003–2006 National Health and Nutrition Examination Survey (NHANES) (29), the 1993–1996 Nutrition and Health Survey in Taiwan (NAHSIT), and the 2005–2008 NAHSIT (30).

Based on the food/beverage items listed in the food frequency questionnaire employed in the NAHSIT (31), the aforementioned 21 food/beverage items were grouped into the following categories: fried/fast food, sweet snack foods (ie, candy, chocolate, cookies, brownies, doughnuts, cakes, pastries, pies, jelly, puddings, sweetened shaved ice desserts or ice cream, popcorn), salty snacks (eg, chips), and sugar-sweetened beverages (ie, carbonated beverages, sugar-added processed juice, lactic acid drinks, sports drinks, instant powered drinks, chocolate beverages, and tea with added-sugar and/or toppings). Each category was analyzed as a separate outcome variable. Because of the right skewed distributions of empty calorie food/beverage consumption variables, we dichotomized these as “none” and “at least one”. Namely, reporting at least one item in a food/beverage category was considered consumption of that food/beverage category during the a given time block. Because salty snack consumption was reported in <5% of the surveys, sweet snack and salty snack consumption were combined as sweet and salty snack food consumption in the analysis.

### Covariates

*Time-varying covariates*. The time-varying covariates in this study included emotions, stress, daily working hours, number of completed EMA surveys per day (range 1–4), and the sequence of EMA survey day (ie, the 1^st^, the 2^nd^, …, or the 14^th^ day).

Emotions were assessed four times per day using the ten-item International Positive and Negative Affect Schedule Short Form (I-PANAS-SF) (32) and four items from the affect subscale of the University of Wales Institute of Science and Technology (UWIST) mood scale (33, 34). In response to the question, “How have you been feeling since the last time you completed a survey?”, items from the I-PANAS-SF (32) were assessed on a five-point Likert scale (ie, never, seldom, sometimes, often, always), while items on the UWIST (33, 34) were assessed on a four-point Likert scale (ie, definitely not, slightly not, slightly, definitely). Measures of positive emotions and negative emotions were constructed by summing scores of the seven positive affect items (range 7–33) and the seven negative affect items (range 7–33), respectively.

Stress was assessed with a single item in the tense arousal UWIST subscale: “stressed” (33, 34). As described above, this item was assessed on a four-point Likert scale from “definitely not” to “definitely”. Daily working hours were assessed based on the record in the published work schedule.

*Time-invariant covariates*. Assessed as part of the baseline survey, time-invariant covariates were person-level measures that included the following: age (in years), gender, educational attainment, marital status, family responsibility (ie, being as a main caregiver for kids or disabled persons in their family), and per capita household income. Other person-level covariates included body mass index (BMI) derived from self-reported height (cm) and weight (kg), health conditions (eg, medical history), smoking and tobacco use, occupational history such as year(s) of working as a registered nurse and history of rotating shift work (in years), and work unit (eg, surgical, medical ward, or intensive care unit).

### Analysis

*Descriptive statistics*. STATA 15 (Statacorp, College Station, TX, USA) was employed for data analysis. Descriptive statistics were employed to describe participants’ characteristics (eg, demographics), daily shift work, momentary empty calorie food/beverage intake (ie, fried/fast food, sweet and salty snacks, and sugar-sweetened beverages), and other time-varying covariates. In addition, momentary empty calorie food/beverage consumption was presented by shift timing: day, evening, or night shift.

*Inferential statistics*. Three-level mixed-effects logistic regression models were employed to test associations between shift work and three categories of empty calorie food/beverage consumption: fried/fast food, sweet and salty snack foods, and sugar-sweetened beverages. We employed backward selection methods based on results of a likelihood ratio test (significance level: 0.05) to select the most parsimonious model for each outcome. If a variable met the criterion for inclusion as a covariate in any model, it was included in models for all outcomes. As a result, the identified moment-level covariates were emotions and stress level; the day-level covariates were the number of complete EMA surveys and the sequence of EMA survey days. The identified person-level covariates were age, BMI, educational attainment, family responsibility, and health conditions.

To test our first hypothesis, we regressed empty calorie food/beverage consumption on same-day shift work (ie, predictor) and the identified time-varying and time-invariant covariates. To test our second hypothesis, we regressed empty calorie food/beverage consumption on prior-day shift timing, same-day shift timing, and the identified covariates.

Mixed-effects regression models are comprised of two components: fixed and random effects. For time varying variables, the effects can be divided into two components: within- and between-person effects (35). In this study, given a sample size of 77, we focused on the within-person effects that captured how changes in a time-varying independent variable in a person (ie, the deviance of person i at time t from the mean level/proportion of person i across time) contributed to that person’s variations in a dependent variable, accounting for that person’s mean level/proportion of that variable across time (35).

### Sensitivity analysis

Our primary strategy involved matching intake in a 24-hour calendar day to shift timing that same calendar day. This approach can capture intake prior to the start of a shift, particularly on evening shifts. However, it is possible that shift work affects subsequent day intake only. As a result, for working days, we tested the sensitivity of results by matching the start of a 24-hour consumption window to the start of a shift. After limiting to those observations that met this criterion, 2031 momentary observations were included in the sensitivity analysis.

It is possible that the effects of shift work on salty snacks differ from its influences on sweet snacks. Therefore, we also tested the sensitivity of results of snack food consumption by removing salty snack consumption from “sweet and salty snack consumption”. The influences of shift work on sweet and salty snacks were analyzed in separate models.

## Results

### Descriptive statistics

After excluding observations with incomplete data, a total of 2444 EMA surveys nested within 961 person-day observations and 77 participants were included. Overall, each participant was followed for 12.5, standard deviation (SD) 2.3 days. The average number of EMA surveys per person-day was 2.5 (SD 1.0). Sample characteristics are presented in table 1. Out of the 961 person-day observations, 23.8% were day shifts, 22.0% were evening shifts, 22.3% were night shifts, and 32.0% were off-duty days. The average hours per working day were 8.2 (SD 0.4) hours. As shown in table 2, participants reported consuming fried/fast food at 16.1% of the signals, sweet and salty snack foods at 32.2% of signals, and sugar-sweetened beverages at 34.8% of signals.

**Table 1:** Participants’ characteristics (N=77). [N=number of participants; N_d_=number of day-level observations; N_m_=number of momentary observations; SD=standard deviation; RN=registered nurse.]

Variables	Mean (SD)	Range	N (%)
Demographic data			
Age (in years)	27.9 (4.5)	22.5–41.9	
Female			73 (94.8)
Married			9 (11.7)
Bachelor’s degree or higher			65 (84.4)
Taking care of kids/disabled people			12 (15.6)
Per capita household income ^a^ (N=75)			
Low (<US$4955)			23 (29.9)
Middle (US$4955–7433)			17 (22.1)
Above middle (>US$7433)			35 (45.5)
Body mass index (kg/m^2^)	23.1 (5.0)	17.2–39.1	
Having ≥1 chronic condition ^b^			18 (23.4)
Current smoker			2 (2.6)
Occupational history (in years)			
Working as an RN	5.7 (4.2)	0.5–20	
Working in rotating shift work	5.4 (4.2)	0.5–20	
Work characteristics			
Working in intensive care units			40 (52.0)
Moment level covariates (N_m_= 2444)			
Emotions			
Positive affect	18.9 (6.2)	7–33	
Negative affect	11.2 (4.5)	7–32	
Experienced stress			827 (37.9)

a Two participants refused to report their annual household income. Per capita income was grouped based on the announcement from the Department of Social Assistance and Social Work, Ministry of Health and Welfare (2019).

b Chronic conditions included diabetes (type 1 or type 2) or high blood sugar, heart diseases (eg, coronary artery disease, angina, congestive heart failure), hypertension, stroke, high cholesterol/hyperlipidemia, thyroid problems (eg, hyperthyroidism, hypothyroidism), kidney diseases (eg, chronic renal failure), cancer or a malignant tumor (excluding minor skin cancer), digestive problems (such as ulcer, colitis, or gallbladder disease), mental illnesses (eg, depression, anxiety), and sleep problems (eg, insomnia).

**Table 2:** Empty calorie food/beverage consumption by shift work at the momentary level (N=77, number of momentary observations=2444) ^a^.

	Overall N=2444	Shift work schedule		

		Day N=584	Evening N=494	Night N=528

	N (%)	N (%)	N (%)	N (%)
Empty calorie food/beverage consumption (any versus none)				
Fried food or fast food	394 (16.1)	67 (11.5)	82 (16.6)	95 (18.0)
Sweet and salty snacks	788 (32.2)	149 (25.5)	159 (32.2)	194 (36.7)
Sugar-sweetened beverages ^b^	850 (34.8)	187 (32.0)	163 (33.0)	182 (34.5)

a 77 participants completed 2444 ecological momentary assessment (EMA) surveys during the 14-day data collection period. Of 2444 observations, 838 were reported on off-duty days, which are not shown.

b When further examining the proportion of caffeinated drinks (eg, cola, tea), we observed that 79.5% (676 out of 850) of sugar-sweetened beverage consumption were caffeinated.

### Inferential statistics

Table 3 shows crude and adjusted associations between shift work and same-day empty calorie food/beverage consumption. After adjusting for covariates and accounting for a person’s usual conditions of shift work, working night shifts was associated with a 70% increase in the likelihood of fried/fast food consumption [adjusted odds ratio (OR_adj_) 1.7, 95% confidence interval (CI) 1.2–2.6] and a 50% increase in the likelihood of sugar-sweetened beverage consumption (OR_adj_ 1.5, 95% CI 1.0–2.1).

**Table 3:** Associations between shift work and same day empty calorie food/beverage consumption ^a^ using mixed-effects regression models (N=77, number of momentary observations=2444). [OR=odds ratio from 3-level mixed-effects logistic regression models; CI=confidence interval.]

Variables	Fried/ fast food		Sweet and salty snacks		Sugar-sweetened beverages	
		
	OR	95% CI	OR	95% CI	OR	95% CI
Crude models						
Shift work ^b^						
Day (reference)						
Evening	1.4	0.9–2.0	1.4	1.0–1.9 ^c^	1.2	0.9–1.7
Night	1.7	1.1–2.5 ^c^	1.3	1.0–1.8	1.3	0.9–1.9
Adjusted models ^d^						
Shift work						
Day (reference)						
Evening	1.3	0.9–1.9	1.3	1.0–1.8	1.1	0.8–1.6
Night	1.7	1.2–2.6 ^e^	1.3	1.0–1.8	1.5	1.0–2.1 ^c^

a The results on the off-duty days were not present in the table.

b Only within-person effects were presented in the table. The within-person effect captured how changes in shift timing given a person contributed to that person’s variations in same-day empty calorie food/beverage consumption.

c P< 0.05.

d The time-varying covariates [ie, emotions, experienced stress, the number of complete ecological momentary assessment (EMA) surveys per day, the sequence of the EMA survey days] and time-invariant covariates (ie, age, body mass index, educational attainment, family responsibility, health conditions) were controlled in the respective models.

e P< 0.01.

Table 4 shows crude and adjusted associations between shift timing and subsequent-day empty calorie food/beverage consumption. In the crude and adjusted models, shift work was not significantly associated with the likelihood of subsequent-day empty calorie food/beverage consumption.

**Table 4:** Associations between shift work and subsequent-day empty calorie food/beverage consumption ^a^ Using mixed-effects regression models (N=77, number of momentary observations=2444). [OR=odds ratio from 3-level mixed-effects logistic regression models; CI=confidence interval.]

Variables	Fried/ fast food		Sweet and salty snacks		Sugar-sweetened beverages	
		
	OR	95% CI	OR	95% CI	OR	95% CI
Crude models						
Shift work on the previous day ^b^					
Day (reference)						
Evening	1.2	0.8–1.7	1.1	0.8–1.6	0.9	0.7–1.3
Night	1.0	0.7–1.5	1.3	1.0–1.8	1.1	0.8–1.5
Adjusted models						
Model 1 ^c^						
Shift work on the previous day						
Day (reference)						
Evening	1.2	0.8–1.7	1.1	0.8–1.5	0.9	0.6–1.3
Night	1.1	0.7–1.6	1.3	1.0–1.8	1.1	0.8–1.6
Model 2 ^d^						
Shift work on the previous day						
Day (reference)						
Evening	1.0	0.6–1.5	0.9	0.7–1.3	0.7	0.5–1.1
Night	0.8	0.5–1.2	1.2	0.9–1.7	0.9	0.6–1.4

a The results on the off-duty days were not present in the table.

b Only within-person effects were presented in the table. The within-person effect examined whether changes in shift timing given a person contributed to that person’s variations in subsequent-day empty calorie food/beverage consumption.

c The identified time-varying [ie, emotions, experienced stress, the number of complete ecological momentary assessment (EMA) surveys per day, the sequence of the EMA survey days] and time-invariant covariates (ie, age, body mass index, educational attainment, family reponsibility, health conditions) were controlled in the respective models.

d In addition to the variables controlled in the Model 1, same-day shift timing was controlled in the respective final models.

### Sensitivity analysis

Results in the sensitivity analysis were consistent with those presented in table 3 and table 4 (see supplementary material, www.sjweh.fi/show_abstract.php?abstract_id=3903, table S1). Compared to day shifts, the likelihood of fried/fast food consumption and sugar-sweetened beverages intake were higher on night shifts. In terms of sweet and salty snack consumption, the results were similar when salty snacks were removed from the snack food measures (supplementary table S2).

## Discussion

This is the first study to examine dynamic within-person associations between shift work and workers’ empty calorie food/beverage consumption. We found that working night shift versus day shift was associated with higher likelihoods of reporting same-day fried/fast food consumption and sugar-sweetened beverage consumption. No associations were found between empty calorie food/beverage consumption and prior-day shift work.

Consistent with de Assis et al’s findings (17), we did not find significant influences of evening shifts on empty calorie food/beverage consumption. Our findings suggested that, on night-shift-working days, the likelihood of fried/fast food consumption and sugar-sweetened beverage intake were higher than on day-shift-working days. There are multiple possible explanations for this finding. First, working night shift is often contrary to a person’s biological clock, which may result in increased risk of circadian disruption (7) and further contribute to imbalanced secretion of appetite-hormones, such as decreased leptin (7, 36) or increased ghrelin (7, 37). Decreased leptin and increased ghrelin have been linked to increased appetite and food consumption (38). This may also explain why we did not observe increased empty calorie food/beverage consumption on evening shift compared to days working on day shift because circadian disruption is less likely.

Second, the food environment (eg, food availability) has been linked to a person’s snack food consumption (22). Bonnell et al (16) reported that rotating shift workers consumed a greater proportion of discretionary snack foods on night compared to day shifts. When examining workers’ food choices, accessibility of food was one of the major themes of increased discretionary snack food consumption (16). However, it was noteworthy that there were no significant associations between shift timing and sweet and salty snack consumption in this study. With the exception of fast food restaurants and convenience stores, most food outlets in Taiwan are closed when people are working at night. It is possible that fast/fried food and sugar-sweetened beverages were more accessible on night shifts, relative to sweet and salty snacks. As a result, associations between shift timing and sweet and salty snack consumption were masked. Additional studies considering the food environment may help to understand if the environment may influence the relationships of shift work and daily empty calorie food/beverage consumption.

Third, night shift workers may consume unhealthy foods (eg, sweetened snacks) to stay awake during their night shifts (39, 40). A qualitative study of 12 registered nurses observed that some nurses consumed high sugar foods and sugar-sweetened beverages to prevent or to cope with their fatigue (41). In this study, we observed that 79.5% (676 out of 850) of participants’ sugar-sweetened beverage consumption were caffeinated (eg, cola, tea). It is possible that the increased likelihood of sugar-sweetened beverage consumption in this study was related to participants’ coping strategies for fatigue from night shifts.

Additionally, shift work may reduce workers’ time available for food preparation (16) or consumption (39). Shift workers tend to purchase and consume something that is easy and quick to prepare (16). Therefore, it is also possible that increased consumption of empty calorie foods/beverages on night shifts is related to decreased time availability for food preparation or consumption on night compared to day shifts.

We found no associations between shift work and subsequent day empty calorie food/beverage consumption. To our knowledge, no study has investigated the dynamic effects of shift work on workers’ next-day empty calorie food/beverage consumption. Shift work may disrupt workers’ circadian alignment (7), which may further imbalance the appetite hormones (eg, ghrelin). Previous studies suggested increased pre-prandial levels of ghrelin were associated with greater appetite or food consumption during that meal (38). These findings are consistent with the possibility that the effect of appetite hormones on food intake is transient instead of long-term.

### Implications for interventions

To improve workers’ health behaviors, multi-level interventions (eg, organizational, individual) are recommended (42, 43). At the organizational level, the food environment has been noted as one of the main reasons for unhealthy food consumption among night shift workers (16). Since most food outlets that offer healthier foods at the workplace are closed at night, creating an environment where all workers have greater access to healthier nutrient-dense foods (ie, salad, fruit, non-fat milk) and less access to unhealthy foods or beverages (44) may help to decrease shift workers’ empty calorie food/beverage consumption on days working night shifts.

Because previous studies have suggested that unhealthy food consumption (ie, discretionary snacks, sugar-sweetened beverages) may be a coping strategy for fatigue on night shifts (39–41), reducing fatigue from shift work may be beneficial for shift workers’ dietary quality. At the organizational level, providing workers a proper break during night shifts, adequate staffing, and workload balance are recommended for the management of fatigue (45). At the individual level, programs that help people develop better coping mechanisms for fatigue (eg, regular exercise, combination of sleep hygiene and light exposure education) (46) may reduce shift workers’ fatigue. In addition, shift workers tend to consume caffeine (eg, cola, coffee, tea) to promote alertness on night shifts (45). Therefore, replacing the source of caffeine intake (eg, from cola to non-sweetened tea or coffee) and educating workers about healthier snack foods (eg, nuts, seeds) as an alternative to promote alertness may reduce the possibility of sugar-sweetened beverage consumption on night shifts.

### Strengths and limitations

This study has several strengths. First, this is the first study to examine within-person associations between shift work and empty calorie food/beverage intake. Second, the prospective intensive longitudinal design using EMA methodology helped to assess participants’ empty calorie food/beverage consumption in real-time, which reduced the chance of recall bias and increased ecological validity. Momentary assessments also allowed testing of short-term effects of shift work. For instance, we could examine whether shift work influenced same- or subsequent-day behavior. In addition, records from published work schedules were employed to assess participants’ exposure to shift work, which reduced participants’ recall bias of work schedules.

This study’s limitations should also be considered. First, the findings of this study may not be generalizable to all rotating shift workers due to the use of a convenience sample of nurses. Participants in this study were relatively young. The healthy work effect (ie, workers who cannot adjust to shift work may choose not to work on rotating shifts) (47) might also introduce selection bias and affect the generalizability of the findings.

Second, a food checklist was employed to assess participants’ empty calorie food/beverage consumption instead of using 24-hour dietary recalls or food records, which may also contribute to misleading findings. For instance, people may report the same number of empty calorie food/beverage items; however, the portion size or number of servings they consumed may differ.

Third, to capture each participant’s shift work pattern, each participant was requested to respond to the EMA survey for 14 days, which may increase their burden and result in overall low response rates. The EMA survey response rate was 56.3%, which is lower than preferred (48) and might hinder the validity of study findings, especially when those observations were not missing randomly. Future studies investigating the feasibility and acceptability of an EMA study among shift workers may contribute to the application of EMA methodologies in this population. In addition, it is possible that workers were unable to respond to an EMA survey due to high work demands (49, 50). Workers often eat when they have time during work. Incorporating event-based EMA surveys during working hours and signal-based EMA surveys during non-working hours or off-duty days may help to better capture shift workers’ food intakes and prevent missing surveys during working hours.

However, in this study, completed EMA surveys from participants were relatively evenly distributed across different shift timings (day: 229, evening: 211, night: 214, off-duty: 307), reducing concern that responses were mainly collected during certain types of shift timings. By asking participants’ empty calorie food/beverage consumption since the last time they completed an EMA survey, it may also help to capture participants’ intake if they had missed any EMA surveys.

### Concluding remarks

Findings in this study revealed that shift work might influence same-day empty calorie food/beverage consumption. Shift work is unavoidable for certain types of industries (eg, healthcare, protective services). Thus, interventions that help to prevent unhealthy eating while working night shifts may be beneficial for rotating shift workers’ health.

## Supplementary material

Supplementary material

## References

[1] Tucker P, Folkard S (2012). Working time, health, and safety:A research synthesis paper.

[2] (2007). International Agency for Research on Cancer Shiftwork.

[3] (2015). Eurofound European Working Conditions Survey, Working Time.

[4] National Center for Health Statistics (2015). National Health Interview Survey (NHIS), 2015 Data Release.

[5] Pan A, Schernhammer ES, Sun Q, Hu FB (2011). Rotating night shift work and risk of type 2 diabetes:two prospective cohort studies in women. PLoS Med.

[6] Barbadoro P, Santarelli L, Croce N, Bracci M, Vincitorio D, Prospero E (2013). Rotating shift-work as an independent risk factor for overweight Italian workers:a cross-sectional study. PLoS One.

[7] Bedrosian TA, Fonken LK, Nelson RJ (2016). Endocrine Effects of Circadian Disruption. Annu Rev Physiol.

[8] Nguyen J, Wright KP Jr (2009). Influence of weeks of circadian misalignment on leptin levels. Nat Sci Sleep.

[9] Bescos R, Boden MJ, Jackson ML, Trewin AJ, Marin EC, Levinger I (2018). Four days of simulated shift work reduces insulin sensitivity in humans. Acta Physiol (Oxf).

[10] Figueiro MG (2017). Disruption of Circadian Rhythms by Light During Day and Night. Curr Sleep Med Rep.

[11] Kramer CK, Swaminathan B, Hanley AJ, Connelly PW, Sermer M, Zinman B (2014). Each degree of glucose intolerance in pregnancy predicts distinct trajectories of β-cell function, insulin sensitivity, and glycemia in the first 3 years postpartum. Diabetes Care.

[12] Tabák AG, Jokela M, Akbaraly TN, Brunner EJ, Kivimäki M, Witte DR (2009). Trajectories of glycaemia, insulin sensitivity, and insulin secretion before diagnosis of type 2 diabetes:an analysis from the Whitehall II study. Lancet.

[13] Nicklas TA, O'Neil CE (2015). Development of the SoFAS (solid fats and added sugars) concept:the 2010 Dietary Guidelines for Americans. Adv Nutr.

[14] McCarthy SN, Robson PJ, Livingstone MB, Kiely M, Flynn A, Cran GW (2006). Associations between daily food intake and excess adiposity in Irish adults:towards the development of food-based dietary guidelines for reducing the prevalence of overweight and obesity. Int J Obes.

[15] Papier K, D'Este C, Bain C, Banwell C, Seubsman S, Sleigh A (2017). Consumption of sugar-sweetened beverages and type 2 diabetes incidence in Thai adults:results from an 8-year prospective study. Nutr Diabetes.

[16] Bonnell EK, Huggins CE, Huggins CT, McCaffrey TA, Palermo C, Bonham MP (2017). Influences on dietary choices during day versus night shift in shift workers:A mixed methods study. Nutrients.

[17] de Assis MA, Kupek E, Nahas MV, Bellisle F (2003). Food intake and circadian rhythms in shift workers with a high workload. Appetite.

[18] Yoshizaki T, Komatsu T, Tada Y, Hida A, Kawano Y, Togo F (2018). Association of habitual dietary intake with morningness-eveningness and rotating shift work in Japanese female nurses. Chronobiol Int.

[19] Mashhadi NS, Saadat S, Afsharmanesh MR, Shirali S (2016). Study of association between beverage consumption pattern and lipid profile in shift workers. Diabetes Metab Syndr.

[20] Bonham MP, Bonnell EK, Huggins CE (2016). Energy intake of shift workers compared to fixed day workers:A systematic review and meta-analysis. Chronobiol Int.

[21] Shan Z, Li Y, Zong G, Guo Y, Li J, Manson JE (2018). Rotating night shift work and adherence to unhealthy lifestyle in predicting risk of type 2 diabetes:results from two large US cohorts of female nurses. BMJ.

[22] Elliston KG, Ferguson SG, Schüz N, Schüz B (2017). Situational cues and momentary food environment predict everyday eating behavior in adults with overweight and obesity. Health Psychol.

[23] Zenk SN, Horoi I, McDonald A, Corte C, Riley B, Odoms-Young AM (2014). Ecological momentary assessment of environmental and personal factors and snack food intake in African American women. Appetite.

[24] Cain SW, Filtness AJ, Phillips CL, Anderson C (2015). Enhanced preference for high-fat foods following a simulated night shift. Scand J Work Environ Health.

[25] Bolger N, Laurenceau JP (2013). Intensive longitudinal methods:an introduction to diary and experience sampling research.

[26] Ministry of Health and Welfare (2017). List of accredited hospitals 2013-2016.

[27] Shiffman S, Stone AA, Hufford MR (2008). Ecological momentary assessment. Annu Rev Clin Psychol.

[28] McMenamin TM (2007). A time to work:recent trends in shift work and flexible schedules. Statistics BoL.

[29] Huth PJ, Fulgoni VL, Keast DR, Park K, Auestad N (2013). Major food sources of calories, added sugars, and saturated fat and their contribution to essential nutrient intakes in the U.S. diet:data from the National Health and Nutrition Examination Survey (2003-2006). Nutr J.

[30] Wu SJ, Pan WH, Yeh NH, Chang HY (2011). Trends in nutrient and dietary intake among adults and the elderly:from NAHSIT 1993-1996 mto 2005-2008. Asia Pac J Clin Nutr.

[31] Lo YL, Hsieh YT, Hsu LL, Chuang SY, Chang HY, Hsu CC (2017). Dietary Pattern Associated with Frailty:Results from Nutrition and Health Survey in Taiwan. J Am Geriatr Soc.

[32] Thompson ER (2007). Development and Validation of an Internationally Reliable Short-Form of the Positive and Negative Affect Schedule (PANAS). J Cross Cult Psychol.

[33] Johnston D, Bell C, Jones M, Farquharson B, Allan J, Schofield P (2016). Stressors, Appraisal of Stressors, Experienced Stress and Cardiac Response:A Real-Time, Real-Life Investigation of Work Stress in Nurses. Ann Behav Med.

[34] Matthews G, Jones DM, Chamberlain AG (1990). Refining the measurement of mood:The UWIST Mood Adjective Checklist. Br J Psychol.

[35] Hedeker DR, Gibbons RD (2006). Longitudinal data analysis.

[36] McHill AW, Melanson EL, Higgins J, Connick E, Moehlman TM, Stothard ER (2014). Impact of circadian misalignment on energy metabolism during simulated nightshift work. Proc Natl Acad Sci USA.

[37] Schiavo-Cardozo D, Lima MM, Pareja JC, Geloneze B (2013). Appetite-regulating hormones from the upper gut:disrupted control of xenin and ghrelin in night workers. Clin Endocrinol (Oxf).

[38] Klok MD, Jakobsdottir S, Drent ML (2007). The role of leptin and ghrelin in the regulation of food intake and body weight in humans:a review. Obes Rev.

[39] Persson M, Mårtensson J (2006). Situations influencing habits in diet and exercise among nurses working night shift. J Nurs Manag.

[40] Tepas DI, Tepas DI (1990). Do eating and drinking habits interact with work schedule variables?. Work Stress.

[41] Gifkins J, Johnston A, Loudoun R (2018). The impact of shift work on eating patterns and self-care strategies utilised by experienced and inexperienced nurses. Chronobiol Int.

[42] Levy DE, Gelsomin ED, Rimm EB, Pachucki M, Sanford J, Anderson E (2018). Design of ChooseWell 365:randomized controlled trial of an automated, personalized worksite intervention to promote healthy food choices and prevent weight gain. Contemp Clin Trials.

[43] Ward DS, Vaughn AE, Hales D, Viera AJ, Gizlice Z, Bateman LA (2018). Workplace health and safety intervention for child care staff:Rationale, design, and baseline results from the CARE cluster randomized control trial. Contemp Clin Trials.

[44] Lowden A, Moreno C, Holmbäck U, Lennernäs M, Tucker P (2010). Eating and shift work - effects on habits, metabolism and performance. Scand J Work Environ Health.

[45] Caldwell JA, Caldwell JL, Thompson LA, Lieberman HR (2019). Fatigue and its management in the workplace. Neurosci Biobehav Rev.

[46] Richter K, Acker J, Adam S, Niklewski G (2016). Prevention of fatigue and insomnia in shift workers-a review of non-pharmacological measures. EPMA J.

[47] McMichael AJ (1976). Standardized mortality ratios and the “healthy worker effect“:scratching beneath the surface. J Occup Med.

[48] Stone AA, Shiffman S (2002). Capturing momentary, self-report data:a proposal for reporting guidelines. Ann Behav Med.

[49] McIntyre TM, McIntyre SE, Barr CD, Woodward PS, Francis DJ, Durand AC (2016). Longitudinal study of the feasibility of using ecological momentary assessment to study teacher stress:objective and self-reported measures. J Occup Health Psychol.

[50] Rutledge T, Stucky E, Dollarhide A, Shively M, Jain S, Wolfson T (2009). A real-time assessment of work stress in physicians and nurses. Health Psychol.

