# Supplementary material for: Shift work relationships with same- and subsequent-day empty calorie food and beverage consumption
Source: Scand J Work Environ Health. 2020 Oct 30;46(6):579–88. doi: 10.5271/sjweh.3903 (PMC7737792; doi:10.5271/sjweh.3903)
Supplement: Supplementary material [file SJWEH-46-579-S001.pdf]

Shift work relationships with same- and subsequent-day empty calorie food and beverage consumption[1]

by Ting-Ti Lin, PhD,[2] Chang Park, PhD, Mary C Kapella, PhD, Pamela Martyn-Nemeth, PhD, Lisa Tussing-Humphreys, PhD, Kathleen M Rospenda, PhD, Shannon N Zenk, PhD

1. *Supplementary tables*
2. *Correspondence to: Ting-Ti Lin, School of Nursing, National Defense Medical Center, No. 161, Sec. 6, Minquan E Rd., Neihu District, Taipei City, 11490, Taiwan. [E-mail: tlin@mail.ndmctsgh.edu.tw].*

Table S1. Associations between Shift Timing and Empty Calorie Food/Beverage Consumption Using Mixed-effects Regression Models (N=77, n_m= 2,031)[a]

	Fried food/ fast food		Sweet and salty snacks		Sugar-sweetened beverages	
	**OR**	**(95% CI)**	**OR**	**(95% CI)**	**OR**	**(95% CI)**
**Hypothesis 1**						
Shift timing						
Day (reference)						
Evening	1.3	(0.9-2.0)	1.3	(0.9-1.7)	1.3	(0.9-1.8)
Night	1.9	(1.2-2.8)**	1.3	(0.9-1.8)	1.7	(1.1-2.4)**
**Hypothesis 2**						
Shift timing on the previous day (n_m= 1,418)[1]						
Day (reference)						
Evening	1.1	(0.6-1.9)	0.9	(0.5-1.4)	0.7	(0.4-1.2)
Night	0.8	(0.4-1.4)	1.4	(0.8-2.3)	0.7	(0.4-1.2)

N: number of participants, n_m: number of momentary observations, OR: odds ratio from 3-level mixed-effects logistic regression models, CI: confidence interval.

[a] A total of 2,031 observations and 1,418 observations were included in the sensitivity test for H1 and H2, respectively. Respective outcome variables were tested by the same analytical models as for H1 and H2. The results on the off-duty days were not present in the table.

*p< 0.05, **p< 0.01, ***p< 0.001

Table S2. Associations between Shift Work and Same Day Snack Consumption[a] Using Mixed-effects Regression Models (N=77, n_m= 2,444)

	Sweet snacks		Salty snacks		Sweet and salty snacks	
**Variables**	**OR**	**(95% CI)**	**OR**	**(95% CI)**	**OR**	**(95% CI)**
**Crude models**						
Shift work[b]						
Day (reference)						
Evening	1.4	(1.0-1.9) *	1.2	(0.6-2.4)	1.4	(1.0-1.9)*
Night	1.3	(0.9-1.8)	1.4	(0.7-3.0)	1.3	(1.0-1.8)
**Adjusted models[c]**						
Shift work						
Day (reference)						
Evening	1.3	(1.0-1.8)	1.0	(0.5-2.1)	1.3	(1.0-1.8)
Night	1.3	(1.0-1.8)	1.4	(0.7-2.9)	1.3	(1.0-1.8)

N: number of participants, n_m: number of momentary observations, OR: odds ratio from 3-level mixed-effects logistic regression models, CI: confidence interval.

[a] The results on the off-duty days were not present in the table.

[b] Only within-person effects were presented in the table. The within-person effect captured how changes in shift timing given a person contributed to that person's variations in same-day empty calorie food/beverage consumption.

[c] The time-varying covariates (i.e., emotions, experienced stress, the number of complete EMA surveys per day, the sequence of the EMA survey days) and time-invariant covariates (i.e., age, BMI, educational attainment, family responsibility, health conditions) were controlled in the respective models.

$^{*}p< 0.05$, $^{**}p< 0.01$, $^{***}p< 0.001$
